# NH_4_Cl-induced metabolic acidosis increases the abundance of HCO_3_^−^ transporters in the choroid plexus of mice

**DOI:** 10.3389/fphys.2024.1491793

**Published:** 2024-10-21

**Authors:** Laura Øllegaard Johnsen, Ahmed Sigad, Kathrine Abildskov Friis, Peder Matzen Berg, Helle Hasager Damkier

**Affiliations:** Department of Biomedicine, Faculty of Health, Aarhus University, Aarhus, Denmark

**Keywords:** choroid plexus, cerebrospinal fluid, HCO_3_^−^ transporter, acidosis, SLC4A

## Abstract

Regulation of cerebrospinal fluid (CSF) pH and brain pH are vital for all brain cells. The acute regulation of CSF pH is dependent on the transport of HCO_3_^−^ across the choroid plexus in the brain ventricles. Acute regulation in response to acidosis is dependent on H^+^ export and HCO_3_^−^ import across the plasma membrane. Acute regulation in response to alkalosis is dependent on HCO_3_^−^ export across the plasma membrane. The objective of the study was to investigate the contribution of the Na^+^-dependent HCO_3_^−^ transporters, Ncbe, NBCn1, and NBCe2 to CSF pH regulation during chronic metabolic acidosis in mice. To induce metabolic acidosis, mice received 0.28 M ammonium chloride (NH_4_Cl) in the drinking water for three, five, or seven days. While *in vivo,* CSF pH measurements did not differ, measurements of CSF [HCO_3_^−^] revealed a significantly lower CSF [HCO_3_^−^] after three days of acid-loading. Immunoblotting of choroid plexus protein samples showed that the abundance of the basolateral Na^+^/HCO_3_^−^ transporter, NBCn1, was significantly increased. This was followed by a significant increase in CSF secretion rate determined by ventriculo-cisternal perfusion. After five days of treatment with NH_4_Cl, CSF [HCO_3_^−^] levels were normalized. After the normalization of CSF [HCO_3_^−^], CSF secretion was no longer increased but the abundance of the basolateral Na^+^-dependent HCO_3_^−^ transporters Ncbe and NBCn1 increased. The luminal HCO_3_^−^ transporter, NBCe2, was unaffected by the treatment. In conclusion, we establish that 1) acidotic conditions increase the abundance of the basolateral Na^+^-dependent HCO_3_^−^ transporters in the choroid plexus, 2) NH_4_Cl loading in mice lowers CSF [HCO_3_^−^] and 3) leads to increased CSF secretion likely caused by the increased capacity for transepithelial transport of Na^+^ and HCO_3_^−^ in the choroid plexus.

## Introduction

Regulation of pH in the central nervous system (CNS) is an important aspect for all brain cells (Leusen, 1972; Hladky and Barrand, 2016). Acid-base disturbances in the CNS can affect neuronal function due to the modified protonation of the membrane proteins that can control the electrical properties of the cells among other factors (Balestrino and Somjen, 1988). Interstitial pH is among the most important factors controlling neuronal excitability (Hladky and Barrand, 2016).

The brain is composed of different fluid compartments: the blood, interstitial (or extracellular fluid), intracellular fluid and cerebrospinal fluid. In the brain parenchyma, the blood-brain barrier is generated by the neurovascular unit that consists of the tight endothelial cells of the capillaries, neurons, glia, smooth muscle cells and pericytes (Profaci et al., 2020). The barrier between the blood and the cerebrospinal fluid (CSF) is generated mainly by the choroid plexus in the brain ventricles (Damkier et al., 2013). The choroid plexus is a layer of tight epithelial cells residing on fenestrated capillaries and connective tissue (Damkier et al., 2013). CSF flows through the brain ventricles and enters the subarachnoid space which is the space between the pia and the arachnoid membrane of the brain and spinal cord. From the subarachnoid space, CSF flows along the penetrating arteries of the brain in the periarterial space also known as the Virchow-Robin spaces (Woollam and Millen, 1955). From here, CSF is believed to distribute into the interstitial space and surrounding the brain cells (Iliff et al., 2012).

In the blood, small changes in pH are transient and normally minimized by plasma proteins, whereas larger changes in pH result in alkalosis or acidosis (Siesjo, 1972). Systemic acid-base disturbances in the body are mainly corrected by the lungs and kidneys. The kidneys control plasma HCO_3_^−^ by excreting acids (NH_4_^+^ and titratable acids) and reabsorption of base (HCO_3_^−^). The lungs control pCO_2_ by increasing or decreasing the amount of CO_2_ expired. The ventilatory response is dependent on central chemoreceptors that sense pCO_2_ and H^+^ in the brain interstitial fluid as chemosensitive neurons on the ventral brainstem surface sense pCO_2_ in the arterial blood. Both the blood-brain barrier (BBB) and the BCSFB are highly permeable to CO_2_ but less permeable to H^+^ and HCO_3_^−^. Elevated arterial pCO_2_ results in a concurrent increase in interstitial pCO_2_, which activates the central chemoreceptors (Dean and Nattie, 2010).

Several studies have shown that acute changes in blood pH are immediately transferred to cerebrospinal fluid (CSF) and interstitial fluid pH (Pappenheimer et al., 1965; Kazemi et al., 1967). However, CSF pH normalizes within minutes to hours depending on species despite plasma pH remaining low (Kazemi et al., 1967; Christensen et al., 2018). In dogs, constant inhalation of 10% CO_2_ for 6 hours results in a rapid CSF pH drop, followed by a slow but steady normalization of CSF pH within the 6 hours, despite continued CO_2_ inhalation and low plasma pH (Kazemi et al., 1967). In mice, the response is even faster; inhalation of 5% CO_2_ causes a rapid 0.02 pH unit drop in CSF pH, which normalizes after 30 min in the presence of 5% CO_2_ (Christensen et al., 2018). The fast normalization of pH is surprising because CSF contains much less protein than plasma (BD and Roberts, 2019). Therefore, CSF is much more dependent on other buffering systems such as the CO_2_/HCO_3_^−^ buffer system (Hladky and Barrand, 2016; Kazemi et al., 1967). As the blood-cerebrospinal fluid barrier (BCSFB) is much less permeable to HCO_3_^−^, compared to CO_2_, it must have a large capacity to increase net transport of base in the form of HCO_3_^−^ (Husted and Reed, 1977).

The CSF is primarily produced by the choroid plexus epithelium (CPE) (Damkier et al., 2013). The choroid plexus is a secretory tissue located in each of the four brain ventricles. The CPE resides on a bed of leaky capillaries and connective tissue that together make up the BCSFB.

**FIGURE 1:**
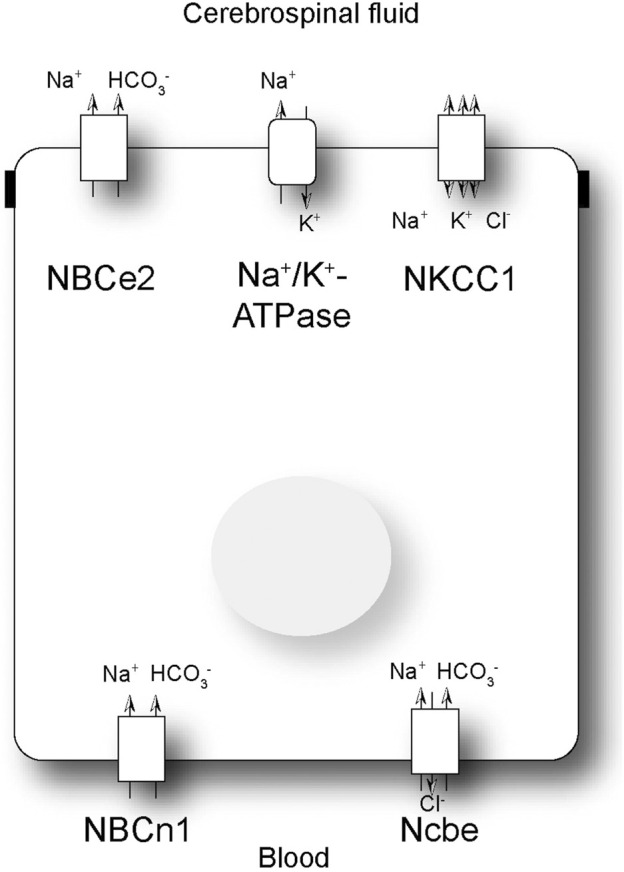
Schematic presentation of a choroid plexus epithelial cell. Schematic presentation of a subset of the transporters involved in net transport of Na^+^ and HCO_3_^−^ across the choroid plexus epithelial cell. NBCe2: electrogenic Na^+^: HCO_3_^−^ cotransporter; NBCn1: electroneutral Na^+^: HCO_3_^−^ cotransporter; Ncbe: electroneutral Na^+^ dependent Cl^−^/HCO_3_^−^ exchanger; NKCC1: Na^+^, K^+^, 2Cl^-^ cotransporter.

The secretion of CSF is known to be dependent on the net transfer of H_2_O, Na^+^, Cl^−^, and HCO_3_^−^ from blood to CSF (Damkier et al., 2013). CSF secretion is dependent of the activity of the Na^+^/K^+^ ATPase, NKCC1 and basolateral bicarbonate transporters (Damkier et al., 2013). Unlike most other epithelia, the CPE cells express the Na^+^/K^+^-ATPase luminally (Masuzawa et al., 1984; Siegel et al., 1984) (Figure 1), actively moving Na^+^ from the cell into CSF in exchange for K^+^. Blocking the Na^+^/K^+^-ATPase with ouabain greatly reduces CSF secretion (Ames et al., 1965). Water is believed to be transported through the water channel AQP1 and paracellularly by claudin-2. AQP1 is very abundant in the luminal membrane of the CPE cells, similar to the proximal tubule of the kidney (Nielsen et al., 1993). The Na^+^, K^+^, 2Cl^-^ cotransporter, NKCC1, is also located in the luminal membrane (Figure 1) (Plotkin et al., 1997; Keep et al., 1994). The transport direction of NKCC1 is, however, currently not completely understood, but it is well-established that NKCC1 is important for CSF secretion, as bumetanide, a NKCC1 inhibitor, reduces CSF secretion (Vogh and Langham, 1981). HCO_3_^−^ is primarily believed to be transported across the luminal membrane via the electrogenic Na^+^/HCO_3_^−^ cotransporter, NBCe2 (Figure 1) (Bouzinova et al., 2005; Millar and Brown, 2008).

In the basolateral membrane of the CPE cells, the primary Na^+^ loader is most likely the Na^+^-dependent Cl^−^/HCO_3_^−^ exchanger, Ncbe (Figure 1) (Praetorius et al., 2004). This transporter is also named NBCn2 as there is a species-dependent variation in the involvement of Cl^−^ transport (Damkier et al., 2010; Parker et al., 2008). Regardless, the transporter is important for transporting Na^+^ as well as HCO_3_^−^ from the blood into the CPE cell. The CPE cells also express the electroneutral Na^+^/HCO_3_^−^ cotransporter, NBCn1 (Bouzinova et al., 2005). The role of NBCn1 in the choroid plexus is not known. Studies have indicated that the transporter can be expressed in both the luminal and basolateral membranes, although the consensus is that it is primarily a basolateral transporter (Figure 1) (Praetorius et al., 2004). Finally, the CPE cells express the anion exchanger AE2 in the basolateral membrane (Alper et al., 1994). AE2 imports Cl^−^ in exchange for HCO_3_^−^, which is important for the net cellular uptake of Cl^−^ and in compensation for alkaline loads.

The role of HCO_3_^−^ transporters in the choroid plexus for CSF pH regulation has not been widely investigated *in vivo* (Christensen et al., 2018). We have previously shown that the luminally located NBCe2 is vital for acute CSF pH regulation (Christensen et al., 2018). In NBCe2 knockout mice, as well as mice with siRNA-mediated NBCe2 knockdown, the recovery of CSF pH after acidification by inhalation of 5% CO_2_ was significantly reduced (Christensen et al., 2018). Ncbe is important for CSF secretion but its role in pH regulation has not been investigated. Knockout of Ncbe results in greatly diminished brain ventricles in mice indicating that the mice have less CSF volume (Jacobs et al., 2008). Interestingly, the knockout mice also show an increased seizure threshold. This could indicate that CSF pH is affected by the deletion of Ncbe, as brain acidosis measured by cortical pH electrodes is known to protect against seizures (Schuchmann et al., 2006).

Our previous studies have investigated the acute effects of acidosis in a respiratory acidosis model. In this study, we investigate the regulation of the Na^+^/HCO_3_^−^ transporter expression in the choroid plexus during experimentally induced chronic metabolic acidosis in adult male C57BL/6J mice. Acidosis was induced by treatment with 0.28 M ammonium chloride (NH_4_Cl) in the drinking water. In the mice subjected to NH_4_Cl treatment, we find an increased abundance of the basolateral Na^+^-dependent HCO_3_^−^ transporters, but not the luminal transporter. Furthermore, we find that three days of NH_4_Cl treatment in the mice result in decreased CSF HCO_3_^−^ levels as well as increased CSF secretion. After longer treatment (five and seven days), HCO_3_^−^ levels normalized, CSF secretion normalized, while the abundance of the basolateral Na^+^-dependent HCO_3_^−^ transporters remained elevated suggesting sustained increased transport of HCO_3_^−^ across the choroid plexus mediated by Ncbe and NBCn1 during chronic acidosis. Our findings suggest an important role of the basolateral choroid plexus transporters, Ncbe and NBCn1, in the regulation of CSF pH during chronic acidosis.

## Methods

### Ethics approval

All animal experiments were conducted in accordance with national guidelines for the care and use of laboratory animals. All experimental protocols were approved within the regulation of the Danish Animal Research Inspectorate and ethical care (Animal experimental license number 2018-15-0201-01526/1 and 2024-15-0201-01662). Ten to 12 weeks old male C57BL/6J mice were fed a standard rodent pellet diet *ad libitum*, had free access to tap water (unless otherwise specified), and were housed in cages containing maximum five mice per cage in a temperature-controlled facility with a 12-h light/dark cycle. All mice were euthanized by cervical dislocation following the completion of the experiments.

### Induction of metabolic acidosis in mice

Metabolic acidosis was induced by providing mice with drinking water containing 0.28 M NH_4_Cl (pH 6.85) similar to (Olsen et al., 2021). Control mice received normal drinking water. During the experimental period, the mice had no access to other water sources but continued to receive food *ad libitum*. As the mice were initially hesitant to drink the ammonium-supplemented water, NH_4_Cl solution was added to a portion of the food (0.28 M NH_4_Cl). The control mice received a similar food portion but with normal drinking water. Mice were weighed daily at the same time each day.

### Blood gas sampling and analysis

Blood samples were obtained from a tail tip amputation from anesthetized mice (ketamine 80 mg/kg and xylazine 10 mg/kg) while the mice received 100% O₂ via a nosecone at a flow rate of 1 L/min. Approximately 60 µL of blood was collected from each mouse. Blood gas analysis was conducted using the ABL90 FLEX PLUS (Radiometer Medical ApS, Brønshøj, Denmark). The machine was calibrated according to the manufacturer’s instructions prior to use. Blood samples were immediately analyzed post-collection to ensure accuracy. Blood was collected in heparinized capillary tubes and inserted directly into the ABL90 FLEX PLUS machine. Parameters measured included pH, partial pressure of oxygen (pO₂), partial pressure of carbon dioxide (pCO₂), base excess, and electrolyte levels (K^+^, Na^+^, Cl^−^) (see Tables 3–5).

### Measurement of *in vivo* lateral ventricle CSF pH

CSF pH was measured similar to a protocol described by (Christensen et al., 2018). Mice were anaesthetized with ketamine (80 mg/kg) and xylazine (10 mg/kg) and deep anaesthesia was confirmed by the absence of toe-pinch reflexes. The anesthetized mouse was then installed in a stereotactic frame, with 100% O₂ delivered via a nose cone at a flow rate of 1 L/min. A small incision was made in the skin on top of the skull to expose bregma. At the coordinates 0.1 mm caudal and 1 mm lateral to bregma, the skull was punctured by a 22-gauge needle. A pH electrode was then inserted 2.5 mm deep into the right lateral brain ventricle through the pre-made hole in the skull. Baseline pH was recorded for 10 min. The pH recordings stabilized after 5 min, and the baseline CSF pH was determined from the final 5 min of the recording. The pH electrode was calibrated after each mouse using a two-point calibration (pH 6.6 and 7.0) in pH buffer.

### CSF sampling and determination of CSF [HCO_3_^−^]

Following the intraventricular CSF pH recordings, CSF samples were collected from the same mice. The anesthetized mouse was again placed in a stereotaxic frame, with 100% O₂ supplied via a nosecone at a flow rate of 1 L/min. The skin and muscles overlying the cisterna magna were carefully dissected. A narrowed capillary tube was inserted through the dura into the cisterna magna, allowing CSF to flow into the capillary. CSF was sampled over a course of 10–15 min. The collected CSF was stored at −20°C until further analysis.

CSF [HCO_3_^−^] was measured as total CO_2_ using a custom-built infrared CO_2_ sensor-based system for small volume samples as previously described (Trepiccione et al., 2017; Berg et al., 2023). Briefly, 1M HCl was added to small-volume CSF samples (4.5–10 µL) in a closed chamber while continuously stirred with a small magnet. The resulting strong acidification converts virtually all HCO_3_^−^ to CO_2_ which was then measured in the air-phase by an infrared CO_2_ sensor (GM70 Carbon Dioxide Meter, Vaisala, Helsinki, Finland). The [HCO_3_^−^] was calculated from linear standard curves generated from known NaHCO_3_ standards (10, 20, and 30 mmol/L). A new standard curve was generated at the start of each session.

### CSF secretion in mice

CSF secretion rate was determined by the ventriculo-cisternal perfusion method, as described in (Oshio et al., 2005). Similar to the pH recordings, mice were deeply anesthetized with ketamine (80 mg/kg) and xylazine (10 mg/kg). Deep sedation was confirmed before the mice were secured in a stereotactic frame with 100% O₂ supplied (1 L/min). Additional bolus anesthesia was administered every 30 min throughout the experiment. The superficial and deep neck muscles were carefully dissected to expose the membrane covering the cisterna magna.

Bregma was located and a cannula connected to an infusion tube was inserted into the right lateral brain ventricle using the same coordinates as previously described for the CSF pH measurements. The infusion tube contained 0.5 µM fluorescent dextran (10,000 MW, Alexa Fluor™ 488) diluted in artificial CSF (aCSF).

A narrowed capillary tube was then inserted through the dura into the cisterna magna. Once CSF was observed in the capillary tube, infusion of the fluorescent dextran through the lateral ventricle was initiated at a rate of 0.7 μL/min using a micro infusion pump (CMA 4004). CSF was continuously collected from the cisterna magna every 20 min for a total of 100 min, with each collection involving the replacement of the capillary tube with a new one. The collected CSF was stored at −20°C protected from light.

CSF secretion rate was calculated using the following equation:

$$V_{f}=r_{i}\left(C_{i}-C_{o}\right)/C_{o}$$

Where V_f_ is CSF formation rate, r_i_ is the infusion rate, C_i_ is the fluorescence of the inflow solution and C_o_ is the fluorescence of the collected CSF from the cisterna magna (outflow). Fluorescence values for C_o_ and C_i_ were determined by diluting samples of the infused aCSF (C_i_) and the collected CSF (C_o_) in a 1:5 ratio with aCSF, followed by fluorescence measurement at 488 nm using a plate reader (EnSpire® Multimode Reader) in a 96-well plate. Samples with visible blood contamination were discarded.

CSF secretion rate was determined as a mean value of the last 40 min of measurements when V_f_ reached a plateau-level.

### Generation of protein samples for immunoblotting

Protein samples were prepared from choroid plexus isolated from the lateral and fourth ventricle from one brain half of each experimental animal. The choroid plexus from the contralateral side of the brain was used for quantitative real-time polymerase chain reaction (RT-qPCR).

To generate protein samples, the pooled choroid plexus was directly dissolved in sample buffer composed of 1.5% (w/vol) sodium dodecyl sulfate (SDS), 40.0 mM 1,4-dithiothreitol (DTT), 6% (v/v) glycerol, 10 mM tris(hydroxymethyl)-aminomethane (Tris), pH 6.8, and bromophenol blue, with an additional 7.5 mg/mL DTT. The samples were then boiled at 95°C for 5 minutes and sonicated three times for five pulses at 20% (Model 150 V/T sonicator, BioLogics Inc., Cary, NC, United States).

To minimize variation in total protein load between samples, proteins were separated by SDS-polyacrylamide gel electrophoresis (SDS PAGE) using a 4%–15% precast polyacrylamide gel (Bio-Rad #5671085) along with a protein marker (Bio-Rad #1610373). The gel was run at a constant voltage of 200 V and stained with Gel Code (Thermo Fisher #24590) for 1 h or InstantBlue™ (ISB1L) for 15 min. For the Gel Code, the gel was de-stained in MiliQ water. The protein bands were visualized using the Invitrogen iBright™ 1500 and protein load was adjusted using ImageJ software.

### Immunoblotting

Proteins were separated by SDS-PAGE in a 4%–15% precast polyacrylamide gel (Bio-Rad #5671085) along with a protein marker (Bio-Rad #1610373). To minimize potential errors due to unequal protein transfer, samples were loaded in pairs, with one treated sample and one control sample per pair. After electrophoresis, proteins were transferred to either a polyvinylidene difluoride (PVDF) or nitrocellulose membrane (for the anti-NBCe2 antibody) using the Trans-Blot turbo system (BioRad #1704273). The transfer was conducted at 25 V and up to 1.0 A for 30 min for PVDF membranes and at 2.5 A and up to 25 V for 7 min for nitrocellulose membranes. The membranes were blocked with 5% skimmed milk in phosphate-buffered saline with Tween® detergent (PBS-T), while membranes used for pNKCC1 blots were blocked with 5% bovine serum albumin (BSA) in PBS-T. The membranes were then incubated over night at 4°C with specific primary antibodies (Table 1), diluted in primary antibody diluent (2% BSA, 2 µM NaN_3_, 0.1% Tween in 0.1M PBS). After primary antibody incubation, the membranes were washed in PBS-T and incubated with appropriate horseradish peroxidase (HRP)-conjugated secondary antibodies (Invitrogen) in 5% skim milk for 1 hour at room temperature. The blots were developed using SuperSignal™ West Femto Maximum Sensitivity Substrate (ThermoFisher Scientific, #34094). The blots were imaged using the Invitrogen iBright 1500 and densitometric analysis was performed using ImageJ software. A cropped image of the specific bands is shown. The full images can be found in the Supplementary Figure.

**TABLE 1:** List of antibodies.

Target	Antibody no.	Host	Source
Ncbe	1139AP	Rabbit	Praetorius et al. (2004)
NBCe2	5558AP	Rabbit	Christensen et al. (2018)
NBCn1	2977AP	Rabbit	Damkier et al. (2006)
NKCC1	Ab59791	Rabbit	Abcam
Na^+^/K^+^-ATPase	3B-0/56–0	Mouse	Kashgarian et al. (1985)
Proteasome 20S (H-120)	sc-67339	Rabbit	Santa Cruz
β-actin	AB8229	Goat	Abcam

### Reverse transcription-polymerase chain reaction

Choroid plexus isolated from the lateral and fourth ventricles of the experimental animals was stored in RNAlater® (Sigma, R0901) until further processing. Total RNA was extracted using the RiboPure™ RNA Purification Kit (Ambion™, AM 1924) according to the manufacturer’s instructions. RNA yield and purity were assessed with a NanoPhotometer® (Implen, GmbH). The samples were treated with DNase (Invitrogen, 18068015) to remove any residual DNA, and complementary DNA (cDNA) was synthesized using the SuperScript II Reverse Transcriptase system (Invitrogen, 18064), according to the manufacturer’s instructions. RT-negative samples served as an internal control for the DNase treatment. Quantitative real-time PCR (RT-qPCR) was performed using the LightCycler® 480 SYBR Green I Master system (Roche, 04707516001) and assessed on the LightCycler® 480 thermal cycler. Each reaction contained 5 µL of the SYBR Green Master Mix, 2 µL of cDNA (approximately 120 ng), 0.5 µL of each primer (10 µM), and nuclease-free water to a final volume of 10 µL. The thermal cycling conditions were as follows: an initial denaturation at 95°C for 5 min, followed by 40 cycles of 95°C denaturation for 10 s, 62°C annealing for 20 s, and 72°C elongation for 30 s.

Primers were designed using Primer-BLAST software (NCBI) and synthesized by Sigma-Aldrich (Merck KGaA). The sequences of the primers used are listed in Table 2. Primer specificity was confirmed by melting curve analysis and agarose gel electrophoresis.

**TABLE 2:** List of primers for qRT PCR.

Gene	Protein	Forward primer (5′-3′)	Reverse primer (5′-3′)	Product size (bp)
Slc4a10	Ncbe	ACACTGAAGACACTGCAGAGCA	AGCGTGTTCCACCTCTATCCA	144
Slc4a5	NBCe2	CAGACATCGGGAAGTCGGTT	GGGCATGACACAGATGACCA	135
Slc4a7	NBCn1	CACGACGTTCTTTCTGTCTTCA	TGGATCAGTAGGCTCGAACT	190
Rn18s	18S	GGGAGCCTGAGAAACGGC	GGGTCGGGAGTGGGTAATTT	68
Ipo8	Importin8	CACCAGACCATGACAACGTG	TGGGCTTCCATACCGTTCAA	171
Sdha	Sdha	CCTCCTGCTATCCGCTCCTA	GGGGAAACGCAGGTAAGCTA	88

The relative expression levels of target genes were calculated using the 2^-ΔΔCt method, with RN18S, IPO8, and SDHA as the internal controls. All reactions were performed in duplicates.

### Statistical analysis

Statistical analysis was performed using GraphPad Prism. A *p*-value below 0.05 was considered statistically significant, and in all cases, ‘*n*’ denoted biological replicants. All data were analyzed for normality using the D’Agostino & Pearson test or the Shapiro-Wilk test. For data that were not normally distributed, a Mann-Whitney test was applied.

CSF pH [HCO_3_^−^], mRNA expression, and CSF secretion were analyzed using students’ unpaired t-test comparing treated animals to controls or values before and after treatment. Western blotting abundance was analyzed using students’ paired t-test. The t-test results are reported as the significance value of the test (*p*-value), presented in the format: *p* = significance value. The results are shown as mean ± standard deviation and confidence intervals are given when statistical significance is observed.

## Results

### NH_4_Cl treatment causes acidosis and a compensatory change in [HCO_3_^−^] in CSF in mice

Mice were treated with 0.28 M NH_4_Cl in drinking water to induce metabolic acidosis. The mice were weighed daily, and the percentage of weight loss from day 0 to the last day of experiment was determined. Mice treated for three days lost significantly more weight than the control mice receiving tap water (percentage of weight loss in treated mice: 0.06% ± 0.02%; control group: −0.007% ± 0.03%, *p* < 0.0001, n = 12). Similarly, mice treated for five and seven days lost significantly more weight compared to control mice (percentage of weight loss in mice treated for five days: −1.4% ± 1.3% versus −0.29% ± 0.8% in the control mice, *p* = 0.02, n = 12; percentage of weight loss in mice treated for seven days: 0.006% ± 0.03% versus −0.02% ± 0.01%, *p* = 0.01, n = 8). Although the weight loss in all experimental groups was larger than controls, the percentage of weight loss was not considered large enough to have a major impact on the interpretation of the results.

To confirm the development of acidosis, blood gas analysis was performed. Mice treated with NH_4_Cl developed metabolic acidosis, as pH, base excess, and pCO_2_ were significantly lower in treated mice compared to controls (Table 3). The lower pCO_2_ likely reflects respiratory compensation. Plasma [K^+^] was significantly increased in the acidotic mice, reflecting the movement of K^+^ from intracellular to extracellular fluid as the concentration of H^+^ increase intracellularly. As expected, plasma [Cl^−^] was also significantly increased in the treated mice, while plasma [Na^+^] did not differ between groups.

**TABLE 3:** Blood gas analysis from mice treated with 0.28 M ammonium chloride for three days.

Blood gas parameters	Control	Treated	*p*-value
pH	7.15 ± 0.06	6.99 ± 0.05	<0.0001
pCO_2_ (kPa)	10.8 ± 0.5	9.7 ± 1.2	0.03
pO_2_ (kPa)	19.1 ± 9	14.3 ± 8	0.55
Acid-base status			
Base excess (mmol/L)	−0.43 ± 3.3	−13.9 ± 3.0	<0.0001
*Electrolytes*			
K^+^ (mmol/L)	5.2 ± 0.3	5.9 ± 0.3	0.0009
Na^+^ (mmol/L)	160 ± 2	160 ± 3	0.61
Cl^−^ (mmol/L)	115 ± 4	130 ± 6	<0.0001

**FIGURE 2:**
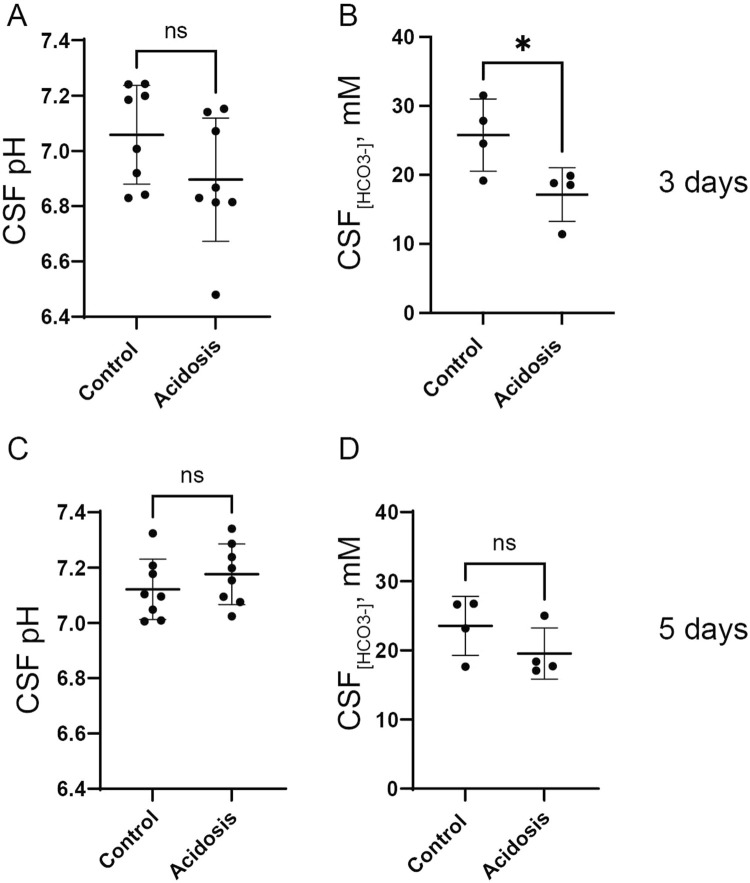
NH_4_Cl treatment transiently decreases CSF [HCO_3_^−^] after 3 days. CSF pH was determined in anaesthetized mice after three **(A)** and five **(C)** days of NH_4_Cl treatment by installation of a micro pH electrode into the right lateral brain ventricle of control and treated mice (acidosis). CSF was extracted from the greater cistern and [HCO_3_^−^] was determined after three **(B)** and 5 days **(D)** of treatment in control and treated mice. Individual values are shown as a scatter plot and supplied with a depiction of mean ± standard deviation. ^*^ indicates *p* < 0.05.

To evaluate the effect of the NH_4_Cl treatment on CSF pH, a pH electrode was inserted directly into the lateral ventricle of anaesthetized mice. After 3 days of NH_4_Cl treatment, CSF pH was tentatively lower in the acidotic mice but not significantly different compared to controls (Figure 2A). CSF has very low levels of protein compared to plasma, and protein normally acutely buffers pH after small pH changes. Therefore, CSF pH regulation depends on the import of HCO_3_^−^ (Damkier et al., 2013). CSF [HCO_3_^−^] was 33% lower in the acidotic mice (Figure 2B), reflecting that CSF [HCO_3_^−^] reacts with protons entering the CSF following the acidotic condition.

Mice treated with NH_4_Cl for five and seven days were also developed metabolic acidosis, as blood pH and base excess were significantly decreased (Tables 4, 5). The difference in base excess diminished as a function of time (13.5 vs 8.3 vs 5.9) reflecting renal compensation. pCO_2_ and plasma [K^+^] did not differ after five- and seven days of treatment, while the increased plasma [Cl^−^] was still observed after both five- and seven days of treatment (Tables 4, 5). Plasma [Na^+^] remained similar between treated mice and control mice at all time points, which confirms no major dehydration despite the small weight loss. CSF pH was similarly measured in mice treated for 5 days, and no statistical difference was found in CSF pH after 5 days (Figure 2C). Unlike after 3 days of treatment, no statistical difference was observed in CSF [HCO_3_^−^] from mice treated for 5 days compared to control (Figure 2D). This result indicates increased HCO_3_^−^ transport into the CSF, compensating for the low level found at the earlier time-point.

**TABLE 4:** Blood gas analysis from mice treated with 0.28 M ammonium chloride for five days.

Blood gas parameters	Control	Treated	*p*-value
pH	7.12 ± 0.05	7.0 ± 0.05	<0.0001
pCO_2_ (kPa)	10.8 ± 1.1	10.5 ± 0.8	0.48
pO_2_ (kPa)	17.4 ± 8	18.8 ± 7	0.66
Acid-base status			
Base excess (mmol/L)	−2.51 ± 2.8	−10.78 ± 4.3	0.0001
*Electrolytes*			
K^+^ (mmol/L)	5.2 ± 0.4	5.4 ± 0.7	0.38
Na^+^ (mmol/L)	156.7 ± 3.0	158.6 ± 3.8	0.18
Cl^−^ (mmol/L)	113 ± 3	122 ± 5	<0.0001

**TABLE 5:** Blood gas analysis from mice treated with 0.28 M ammonium chloride for seven days.

Blood gas parameters	Control	Treated	*p*-value
pH	7.14 ± 0.03	7.04 ± 0.04	<0.0001
pCO_2_ (kPa)	11.3 ± 0.7	12.2 ± 1.4	0.15
pO_2_ (kPa)	27 ± 10	27.7 ± 4.7	0.86
Acid-base status			
Base excess (mmol/L)	0.1 ± 1.5	−5.9 ± 3.8	0.001
*Electrolytes*			
K^+^ (mmol/L)	5.4 ± 0.4	5.5 ± 0.4	0.9
Na^+^ (mmol/L)	157 ± 2	157 ± 2	0.8
Cl^−^ (mmol/L)	110 ± 2	119 ± 4	0.0002

### Three-day NH_4_Cl treatment increases abundance of the basolateral transporter, NBCn1, in the choroid plexus and increases CSF secretion rate

**FIGURE 3:**
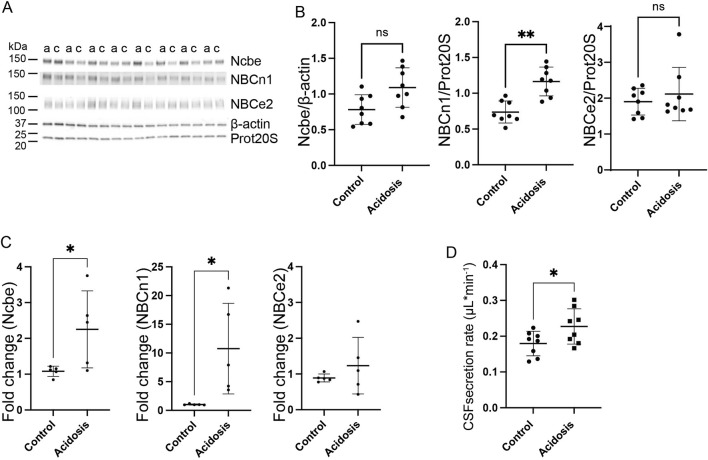
Three days NH_4_Cl treatment increases abundance of choroid plexus basolateral HCO_3_^−^ transporters and augments CSF production rate. Mice received 0.28 M NH_4_Cl in the drinking water for 3 days. Choroid plexus protein samples were subjected to immunoblotting for Ncbe, NBCn1, and NBCe2 **(A, B)**. The specific bands for each protein and either proteasome 20S (Prot20S) or β-actin were detected and quantified densiometrically comparing protein samples from treated **(A)** and control **(C)** mice. Scatter plots show the expression of Ncbe relative to β-actin after 3 days (**(B)**, left), NBCn1 relative to proteasome 20S (**(B)**, middle), and NBCe2 relative to proteasome 20S (**(B)**, right) as well as mean ± standard deviation. Choroid plexus RNA was extracted from the contralateral brain hemisphere and cDNA was subjected to qRT-PCR. Scatter plots show the fold change of Ncbe (**(C)**, left), NBCn1 (**(C)**, middle), and NBCe2 (**(C)**, right) as well as mean ± standard deviation. CSF production rate **(D)** was determined by ventriculo-cisternal perfusion. Scatter plots show the individual CSF production rates (μL/min) and mean ± standard deviation in treated (acidosis) and control mice. ^*^indicates *p* < 0.05; ^**^indicates *p* < 0.01, n. s. no statistical significance.

The choroid plexus expresses three Na^+^-dependent HCO_3_^−^ transporters capable of net HCO_3_^−^ transport in the direction from blood to CSF. Immunoblotting of choroid plexus isolated from mice showed that the basolaterally expressed Na^+^ dependent Cl^−^/HCO_3_^−^ exchanger, Ncbe, did not change abundance in the 3-day NH_4_Cl-treated mice compared to controls that received normal tap water (Figures 3A, B). However, the abundance of the electroneutral Na^+^:HCO_3_^−^ cotransporter, NBCn1, was increased by 37% (95% CI of diff [-0.6 to −0.2], *p* = 0.0015, n = 8, Figures 3A, B). after 3 days of NH_4_Cl treatment. RT-qPCR on choroid plexus from mice subjected to NH_4_Cl treatment showed that mRNA levels of Ncbe and NBCn1 was significantly increased after 3 days of treatment. Ncbe mRNA expression was increased by 108.4% (t (8) = 2.411, 95% CI [0.05118 to 2.291], *p* = 0.0424, n = 5, Figure 3C left) and NBCn1 expression was increased by 948.8% (t (8) = 2.757), 95% CI [1.591 to 17.87], *p* = 0.0248, n = 5, Figure 3C middle). The abundance of the luminally expressed electrogenic Na^+^:HCO_3_^−^ cotransporter, NBCe2, however, remained unaltered both at the protein (Figures 3A, B) and RNA levels (Figure 3C right).

The Na^+^-dependent HCO_3_^−^ transporters are not only involved in the transport of HCO_3_^−^ but also transport Na^+^. The production of CSF is known to be dependent on the net movement of both Na^+^ and HCO_3_^−^. To determine whether the altered abundance of the HCO_3_^−^ transporters could influence CSF production, the CSF secretion rate was determined by ventriculo-cisternal perfusion. Indeed, CSF secretion rate was increased by 21% (95% CI of diff [0.002 to 0.09], *p* = 0.04, n = 8, Figure 3D). in treated mice compared to control mice.

CSF secretion is driven by the activity of the luminal Na^+^/K^+^-ATPase as well as activity of the luminally expressed Na^+^, K^+^, 2Cl^-^ cotransporter, NKCC1. The abundance of neither of these proteins was, however, increased (Supplementary Figure S1A, B).

### The basolateral HCO_3_^−^ transporters, Ncbe and NBCn1, remain increased after five and 7 days of NH_4_Cl treatment without affecting CSF secretion rate

**FIGURE 4:**
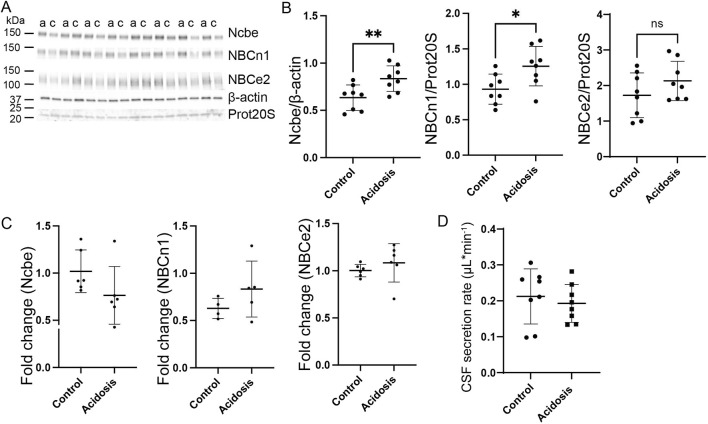
Abundance of basolateral HCO_3_^−^ transporters sustain abundance increase after 5 days NH_4_Cl treatment while CSF secretion is normalized. Mice received 0.28 M NH_4_Cl in the drinking water for 5 days. Choroid plexus protein samples were subjected to immunoblotting for Ncbe, NBCn1, and NBCe2 **(A, B)**. The specific bands for each protein and either proteasome 20S (Prot20S) or β-actin were detected and quantified densiometrically comparing protein samples from treated **(A)** and control **(C)** mice. Scatter plots show the expression of Ncbe relative to β-actin after 5 days (**(B)**, left), NBCn1 relative to proteasome 20S (**(B)**, middle), and NBCe2 relative to proteasome 20S (**(B)** right) as well as mean ± standard deviation. Choroid plexus RNA was extracted from the other brain half and cDNA was subjected to qRT-PCR. Scatter plots show the fold change of Ncbe (**(C)**, left), NBCn1 (**(C)**, middle), and NBCe2 (**(C)**, right) as well as mean ± standard deviation. CSF production rate **(D)** was determined by ventriculo-cisternal perfusion. Scatter plots show the individual CSF production rates (μL/min) and mean ± standard deviation in treated (acidosis) and control mice. ^*^indicates *p* < 0.05, n. s. no statistical significance.

Western blotting of choroid plexus isolated from mice treated with NH_4_Cl for 5 days, showed that the abundance of Ncbe was significantly increased by 24% (95% CI of diff [-0.3 to −0.09], *p* = 0.004, n = 8, Figures 4A, B). NBCn1 abundance also increased by 16% (95% CI of diff [-0.6 to −0.08], *p* = 0.02, n = 8, Figures 4A, B). However, the abundance of the luminally located electrogenic Na^+^:HCO_3_^−^ cotransporter, NBCe2, did not differ between the control group and the mice that were treated for 5 days (Figures 4A, B). Despite the continued increase in protein abundance, mRNA expression levels had normalized to control levels for all transporters after 5 days of treatment (Figure 4C).

Given the increased abundance of the basolateral Na⁺ transporters after 5 days, it was expected that CSF secretion might be affected similarly to what was observed after 3 days of treatment. However, no difference in CSF secretion rate was observed (Figure 4D).

Protein abundance of the Na^+^/K^+^-ATPase and NKCC1 was also investigated after 5 days of treatment. The abundance of the Na^+^/K^+^-ATPase was unaffected by treatment (Supplementary Figure S1), but the abundance of NKCC1 was increased by 18% (95% CI of diff [-0.5 to-0.04], *p* = 0.03, n = 8, Supplementary Figure S1C, D).

**FIGURE 5:**
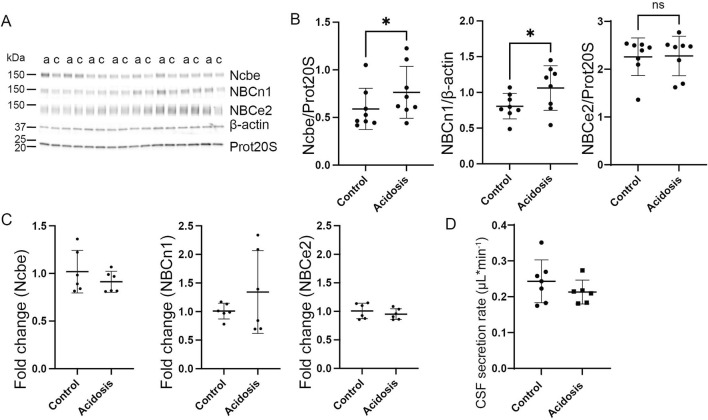
Prolonged increase in abundance of basolateral HCO_3_^−^ transporters after 7 days of NH_4_Cl treatment. Mice received 0.28 M NH_4_Cl in the drinking water for 7 days. Choroid plexus protein samples were subjected to immunoblotting for Ncbe, NBCn1, and NBCe2 **(A, B)**. The specific bands for each protein as well as proteasome 20S (Prot20S) and β-actin were detected and quantified densiometrically comparing protein samples from treated **(A)** and control **(C)** mice. Scatter plots show the expression of Ncbe relative to proteasome 20S (**(B)**, left), NBCn1 relative to β-actin (**(B)**, middle), and NBCe2 relative to proteasome 20S (**(B)** right) as well as mean ± standard deviation. Choroid plexus RNA was extracted from the contralateral side of the brain and cDNA was subjected to qRT-PCR. Scatter plots show the fold change of Ncbe (**(C)**, left), NBCn1 (**(C)**, middle), and NBCe2 (**(C)**, right) as well as mean ± standard deviation. CSF production rate **(D)** was determined by ventriculo-cisternal perfusion. Scatter plots show the individual CSF production rates (μL/min) and mean ± standard deviation in treated (acidosis) and control mice. ^*^indicates *p* < 0.05, n. s. no statistical significance.

The effect of acidosis on the HCO_3_^−^ transporters persisted after 7 days of treatment. The abundance of Ncbe remained increased by 23% (95% CI of diff [-0.3 to −0.03], *p* = 0.03, n = 8, Figures 5A,B). Similarly, the abundance of NBCn1 was increased by 10% (95% CI of diff [-0.5 to −0.03], *p* = 0.03, n = 8, Figures 5A, B). Similar to earlier time points, NBCe2 abundance did not differ between acidotic and control mice after 7 days (Figures 5A, B).

Similar to the levels after 5 days, mRNA expression levels did not differ between treated and control mice (Figure 5C). The CSF secretion rate also was unaffected after 7 days of treatment despite the increased abundance of transporters (Figure 5D).

The protein abundance of the Na^+^/K^+^-ATPase and NKCC1 were both unaffected by 7 days of treatment (Supplementary Figure S1E, F).

## Discussion

The choroid plexus actively participates in the acute regulation of CSF pH during acidotic disturbances by secreting HCO_3_^−^ (Christensen et al., 2018; Husted and Reed, 1977). In mice, CSF pH rapidly drops in response to inhalation of 5% CO_2_. This is followed by a relatively fast normalization of CSF pH dependent on the electrogenic Na^+^:HCO_3_^−^ cotransporter, NBCe2 in the choroid plexus, returning to baseline levels within 20 min while continuously inhaling 5% CO_2_ (Christensen et al., 2018).

To our knowledge, this is the first study to investigate the role of the choroid plexus transporters in CSF pH regulation in a metabolic acidosis model. We show that mice treated with NH_4_Cl develop metabolic acidosis. We also demonstrate that mouse CSF [HCO_3_^−^] is decreased after 3 days of NH_4_Cl treatment but normalizes to control levels after 5 days. NH_4_Cl treatment increases the abundance of the basolateral Na^+^-dependent HCO_3_^−^ transporters in the choroid plexus, which are expressed in the membrane facing the blood. This suggests an increased capacity for HCO_3_^−^ import into the cells in response to the lowered CSF- or blood [HCO_3_^−^]. The luminal NBCe2 is unaltered at all time-points, confirming its primary role in acute regulation of CSF pH. This raises the question of how HCO_3_^−^ is transported across the luminal membrane if not through NBCe2. Abundance of a transporter is, however, not directly comparable to function. NBCe2 transports with a 1 Na^+^:3 HCO_3_^−^ stoichiometry, giving it a large capacity to transport HCO_3_^−^. Thus, further studies are needed to clarify the role of NBCe2 in chronic acidosis.

The mice were given NH_4_Cl in the drinking water. NH_4_Cl dissociates into NH_4_^+^ and Cl^−^ and the increased ammonium-load will result in acidosis. In the liver, NH_4_^+^ is metabolized to urea at the expense of HCO_3_^−^. During acidosis, the proximal tubules of the kidneys secrete NH_4_^+^/NH_3_ into the filtrate. NH_4_^+^ is transported into the thick ascending limb where it is converted into H^+^ and NH_3_. The basolateral electroneutral Na^+^:HCO_3_^−^ cotransporter, NBCn1 in the thick ascending limb imports HCO_3_^−^, which buffers H^+^ and generates CO_2_ and H_2_O. Increased acid load leads to an increased in NBCn1 abundance in the kidney, facilitating increased intracellular buffering of H^+^, allowing for an increased interstitial ammonium gradient from the cortex to the inner medulla. NH_3_/NH_4_^+^ is then shuttled into the interstitial space, secreted in the colleting duct, and excreted with the final urine in the ammonia shortcut mechanism (Olsen et al., 2021; Odgaard et al., 2004).

During NH_4_Cl treatment, various factors other than pH could influence the response on the choroid plexus transporters such as a change of the brain blood flow, a direct effect of NH_4_Cl on transporters, and perhaps other unknown factors. A previous study suggested a direct effect of ammonium on the Na^+^/K^+^-ATPase in the choroid plexus of rats, similar to that seen in hepatic encephalopathy (Alexander et al., 2005). Another study showed that ammonium acetate treatment resulted in enlarged lateral ventricle choroid plexus and “trafficking” of the water channel, AQP 1, to the luminal membrane (Nakadate and Kamata, 2022). Both studies suggest a direct effect of ammonium on the transporters of the choroid plexus, though their time frames (minutes to hours) were shorter than the present study. NKCC1 can transport NH_4_^+^ (Bergeron et al., 2003), and its role in NH_4_^+^ transport in the choroid plexus could explain the increase in NKCC1 abundance observed in this study.

In this study, three timepoints were chosen to investigate the effect of acidosis on the choroid plexus. While attempting to assess the effects of NH₄Cl treatment at 24 h, we observed a significant weight loss in the mice, likely due to decreased water intake from the taste aversion to NH₄Cl. Eventually, the weight normalized when the mice were habituated to the taste. No effect on renal NBCn1 abundance was observed in the mice treated for 24 h; therefore, the data from these mice were deemed as inconclusive (data not shown).

Our data suggest that lowering CSF [HCO_3_^−^] initially increases the abundance of the basolateral transporters, Ncbe and NBCn1, to facilitate transcellular transport of HCO_3_^−^ and normalize CSF [HCO_3_^−^]. Whether this increased abundance is a result of the low pH in the blood, low [HCO_3_^−^] in the blood, or low CSF [HCO_3_^−^] remains unclear. The fact that only the basolateral transporters are affected could suggest that the signal occurs from the blood side, but this is speculative. Further studies are, therefore, necessary to explore this in detail. How the choroid plexus responds to changes in pH is currently unknown.

We observed an increase in cerebrospinal fluid secretion rate after 3 days of treatment. We interpret this as a response to decreased CSF [HCO_3_^−^]. This likely initiates a signal for the basolateral Na^+^:HCO_3_^−^ cotransporters, with a net direction of Na^+^ and HCO_3_^−^ from blood to CSF, to increase abundance and activity. NBCn1 has not previously been described to play a role in CSF secretion but this study suggests that during acidotic conditions, NBCn1 could participate in the net transport of Na^+^ to drive CSF secretion. Ncbe abundance is not increased at the protein level after 3 days but an increase in mRNA is observed. This suggests that there is a larger requirement for Ncbe activity, and indeed, abundance of Ncbe is increased after 5 days. Unlike NBCn1, Ncbe is known to be an important factor in CSF secretion, as mice lacking Ncbe have smaller brain ventricles (Jacobs et al., 2008). The increase in CSF secretion could therefore be caused by the acutely increased activity of both Ncbe and NBCn1 as a response to the low [HCO_3_^−^]. However, after 5 days, CSF secretion rates normalize despite increased transporter abundance, possibly reflecting reduced transporter activity as mRNA levels return to normal.

In conclusion, we show that the Na^+^-dependent HCO_3_^−^ transporters in the choroid plexus, Ncbe, and NBCn1 increase in abundance during metabolic acidosis. This increases the capacity for the transport of Na^+^ and HCO_3_^−^ transport across the choroid plexus leading to normalization of CSF pH. This study is the first to uncover the regulation of molecular mechanisms in the choroid plexus, that may control CSF pH and [HCO_3_^−^] during chronic acid-base disturbance. Further studies are needed to investigate how the choroid plexus senses that an increase in the molecular machinery driving HCO_3_^−^ secretion is necessary.

## Data Availability

The original contributions presented in the study are included in the article/Supplementary Material, further inquiries can be directed to the corresponding author.
