# Supplementary figures and images for: NH4Cl-induced metabolic acidosis increases the abundance of HCO3 − transporters in the choroid plexus of mice
Source: Front Physiol. 2024 Oct 21;15:1491793. doi: 10.3389/fphys.2024.1491793 (PMC11532781; doi:10.3389/fphys.2024.1491793)

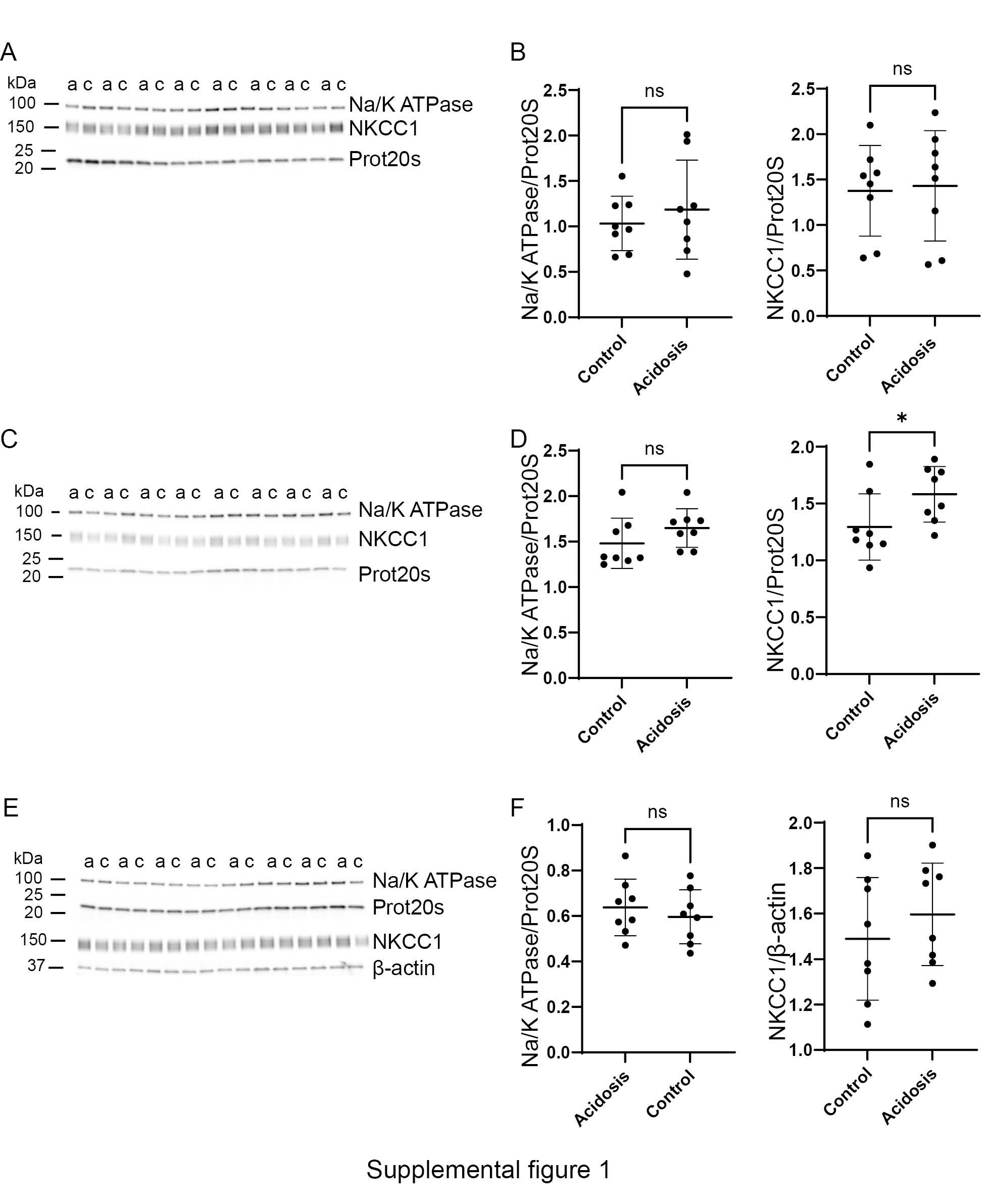

Supplement: Supplementary file 1 [file Image1.JPEG]

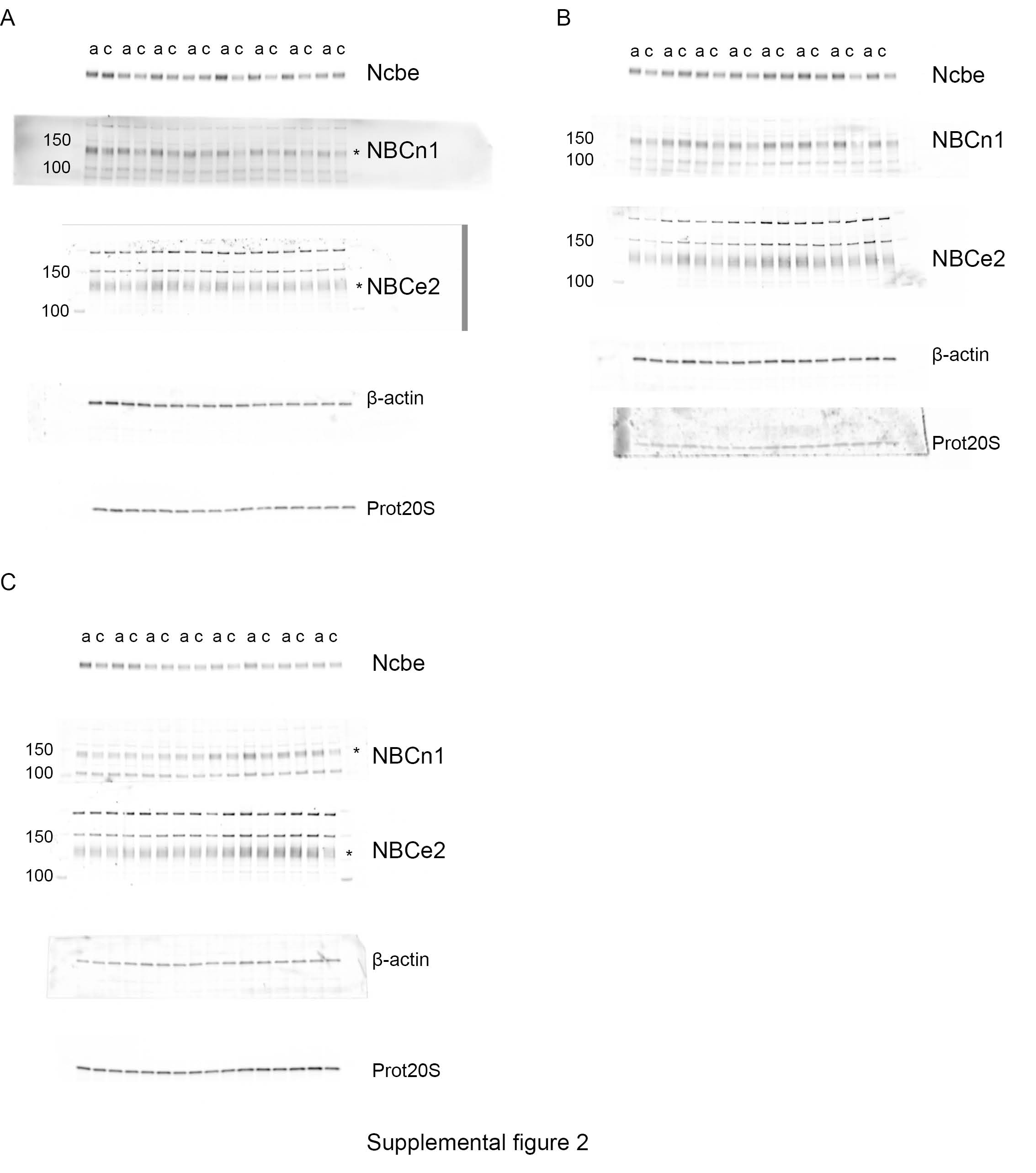

Supplement: Supplementary file 2 [file Image2.JPEG]
