# Supplementary material for: NH4Cl-induced metabolic acidosis increases the abundance of HCO3 − transporters in the choroid plexus of mice
Source: Front Physiol. 2024 Oct 21;15:1491793. doi: 10.3389/fphys.2024.1491793 (PMC11532781; doi:10.3389/fphys.2024.1491793)
Supplement: Supplementary file 3 [file DataSheet1.DOCX]

Supplementary Material

**Supplementary Figure 1.** Mice received 0.28 M NH_4_Cl in the drinking water for three (A + B), five (C+D), and seven (E+F) days. Choroid plexus protein samples were subjected to immunoblotting for the Na^+^/K^+^ ATPase and NKCC1. The specific bands for each protein and either proteasome 20S (Prot20S) or β-actin were detected and quantified densiometrically comparing protein samples from treated (a) and control (c) mice. Scatter plots show the expression of the Na^+^/K^+^ ATPase and NKCC1 relative to proteasome 20s after three days (B) and five days (D) as well as mean ± standard deviation. Scatter plot shows the expression of the Na^+^/K^+^ ATPase relative to proteasome 20S (F, left) and NKCC1 relative to β-actin after seven days (F, right) as well as mean ± standard deviation.

**Supplementary Figure 2.** Uncropped images of western blots shown in figures 2A, 3A and 4A in manuscript from three days (A), five days (B), and seven days (C) from treated (a) and control (c) mice.
